
JOURNAL INFORMATION
==============================
NLM Title Abbreviation: Innov Surg Sci
Journal ID: Innov Surg Sci
NLM Journal ID: 101708165
Journal ID: iss

Innovative Surgical Sciences

EISSN: 2364-7485
Publisher: De Gruyter

ARTICLE INFORMATION
==============================
PMCID: PMC10216889
DOI: 10.1515/iss-2023-9001
Article ID: iss-2023-9001
Article version: 1
Subjects: Article

Abstract DGT

Publication date: 2023 Mar
Electronic publication date: 2023 Mar 13
Volume: 8
Issue: Suppl1
First page: 1
Last page: 9
Copyright: © the Author(s), published by De Gruyter
Copyright year: 2023
Copyright holder: the Author(s), published by De Gruyter
License: This work is licensed under the Creative Commons Attribution 4.0 International License.
License URL: https://creativecommons.org/licenses/by/4.0/

Page count: 9

==============================

**Are routine chest x-rays necessary following thoracic surgery? A systematic literature review and meta-analysis.**

ID: 24

**Kategorie: DGT - Komplikationen in der Thoraxchirurgie (u. a. COVID und Neue Therapien)**

Christian Galata[1], Lorena Cascant Ortolano[2], Saeed Shafiei[1], Svetlana Hetjens[3], Lukas Müller[4], Roland Stauber[5], Davor Stamenovic[1], Eric Rößner[1], Ioannis Karampinis[1]

[1]*Mainz, Klinik und Poliklinik für Thoraxchirurgie, Mainz, Deutschland*, [2]*Mainz, Bereichsbibliothek Universitätsmedizin Mainz, Mainz, Deutschland*, [3]*Medizinische Statistik, Biomathematik und Informationsverarbeitung, Universitätsmedizin Mannheim, Mannheim, Deutschland*, [4]*Mainz, Klinik und Poliklinik für Diagnostische und Interventionelle Radiologie, Mainz, Deutschland*, [5]*Mainz, Hals-, Nasen-, Ohrenklinik und Poliklinik, Molekulare Tumorbiologie, Mainz, Deutschland*

**Background:**

The number of chest x-rays that are performed in the perioperative window of general thoracic surgery varies. Many clinics x-ray patients daily while others only perform x-rays if there are clinical concerns. The purpose of this study was to assess the evidence of perioperative x-rays following thoracic surgery and estimate the clinical value with regards to changes in patient care.

**Materials and methods:**

A systematic literature research was conducted up until November 2021. Studies reporting x-ray outcomes in adult patients undergoing general thoracic surgery were included. Studies in patients undergoing cardiac surgery, esophageal surgery or patients on the intensive care unit were excluded. The search strategy included proper combinations of the MeSH terms ‘x-ray’ and ‘thoracic surgery’.

**Results:**

11 studies (3841 patients/4784 x-rays) were included. The x-ray resulted in changes in patient care in 488 cases (10.74%). In patients undergoing mediastinoscopic lymphadenectomy or thoracoscopic sympathectomy, postoperative x-ray never led to changes in patient care.

**Conclusion:**

There are no data to recommend an x-ray before surgery or to recommend daily x-rays. X-rays that are performed immediately after surgery (post- anaesthesia care unit or recovery) seem to rarely have any consequences. It is probably reasonable to keep requesting x-rays after drain removal since they serve multiple purposes and alter patient care in 7.30% of the cases.

Open Access. © 2023 The Author(s), published by De Gruyter. (cc) BY This work is licensed under the Creative Commons Attribution 4.0 International License.

**The heart is fixed, but the sternum? – Long term outcomes using titanium bar reconstruction for sternal dehiscence after cardiac surgery**
ID: 669

**Kategorie: DGT - Komplikationen in der Thoraxchirurgie (u. a. COVID und Neue Therapien)**

Nora Mayer[1], Murat Kapdagli[1], Fabio De Robertis[1], Jonathan Finch[1], Nizar Asadi[1], Emma Beddow[1], Vladimir Anikin[1]

[1]*Harefield Hospital, Royal Brompton & Harefield Hospitals as Part of Guys & St. Thomas NHS Foundation Trust, London, United Kingdom*

**Background:**
Sternal dehiscence following median sternotomy for cardiac surgery occurs in 0.9 - 5%. Main symptoms are pain and discomfort, clicking sensations and unstable sternum. Sternal repair remains a complex operation for highly selected patients. Titanium bars have been successfully used for sternal reconstruction. Omentoplasty has shown to increase local blood supply and could be beneficial in sternal repair after coronary artery bypass grafting (CABG) with harvesting of bilateral internal thoracic arteries.

**Materials and methods:**
Complex reconstruction for sternal dehiscence was performed in 19 patients (85.7% male) with mean age 63y (SD ± 10) in between 09/2010 to 07/2022. Titanium bar reconstruction plus omentoplasty was done in 84.2% (N=16) patients while 15.8% (N=3) underwent titanium bar fixation only. A standardized surgical approach with fixation of the sternum with titanium bars (The STRAsbourg Thoracic Osteosynthesis System - STRATOS™, MedXpert, Eschbach, Germany) plus median laparotomy with omentoplasty was done.

**Results:**
Patients diagnosed with sterile sternal dehiscence had previously undergone CABG in 73.7% (N=14), CABG + valve replacement in 15.8% (N=3) and valve replacement alone in 10.5% (N=2). Bilateral internal thoracic arteries had been harvested in 42.1% (N=8) of the patients. Mean BMI was 33.1kg/m$^2$ (SD ± 5.2), diabetes mellitus was diagnosed in 36.8% (N=7), 10.5% (N=2) were current smokers, 63.2% (N=12) ex-smokers and COPD was present in N=1 (4.8%). 3 (14.3%) of the patients underwent incisional hernia repair within the initial procedure. Mean LOS was 24 days (SD ± 29.6). No postoperative mortality was observed. Bars were removed electively in 63% (N=12) patients with repair of postoperative symptomatic incisional hernia in 31.6% (N=6) patients. 73.7% (N=14) patients were discharged from our care with stable sternum after a mean follow-up of 48 months (SD ± 60).

**Conclusion:**

Based on our single-centre experience, highly selected and motivated patients benefit from complex STRATOS bar sternal repair and omentoplasty with promising long-term results.

**Current Predictors of Outcome in surgically managed Patients with Parapneumonic Pleural Empyema**
ID: 603

**Kategorie: DGT - Thoraxchirurgie bei Alten und Hochbetagten**

Michael Schweigert[1], Ahmed Hamdouna[1], Attila Dubecz[2], Ana Beatriz Almeida[1], Patrick Kellner[3], Helmut Witzigmann[4], Hubert J. Stein[2]

[1]*Universitätsklinikum Schleswig-Holstein, Campus Lübeck, Klinik für Chirurgie, Lübeck, Deutschland*, [2]*Klinikum Nürnberg, Klinik für Allgemein-, Viszeral- und Thoraxchirurgie, Nürnberg, Deutschland*, [3]*Universitätsklinikum Schleswig-Holstein, Campus Lübeck, Klinik für Anästhesiologie und Intensivmedizin, Lübeck, Deutschland*, [4]*Städtisches Klinikum Dresden-Friedrichstadt, Klinik für Allgemein-, Viszeral- und Thoraxchirurgie, Dresden, Deutschland*

**Background:**
Parapneumonic pleural empyema is a critical condition with substantial morbidity and mortality. Surgical intervention is often complicated by multimorbidity, old age, frailty and advanced septic disease.

**Materials and methods:**
In a retrospective study from a prospective database the outcome of surgically managed patients with parapneumonic pleural empyema at four German tertiary referral hospitals was analyzed. Study period was 2006 – 2021.

**Results:**
There were 571 patients (female 176, male 395). Mean age was 60.57 years. Multimorbidity (mean Charlson Score 2.48) with predominately cardiac conditions (222/571), diabetes mellitus (131/571), neurological disorders (100/571) and COPD (100/571) was common. VATS, open decortication and window thoracostomy were carried out in 437, 120 and 14 cases, respectively. ICU admission (325/571), mean ICU days (11.6), mean LOS (25.3 days) and in-hospital-mortality (47/571) underline the severity of the condition. Sepsis (OR 9.92; 95% CI: 4.99-19.72; p<0.01), respiratory failure (OR 21.47; 95% CI: 10.26-44,98; p<0.01), acute renal failure (OR 9.54; 95% CI: 4.79-18.99; p<0.01), ICU stay > 1 day (OR 13.17; 95% CI: 5.49-31.61; p<0.01) and Charlson Score ≥ 3 (OR 5.34; 95% CI: 2.71-10.55; p<0.01) had higher odds for fatal outcome. Multimorbidity (Charlson Score ≥ 3) was also associated with significantly higher odds for sepsis and ICU admission. In contrast, advanced age (≥ 80 years) was not associated with higher mortality (11/83 vs. 36/488; p=0.08).

**Conclusion:**

Delayed referral for surgery with already advanced septic disease as well as pre-existing multimorbidity are the main reasons for fatal outcome. The most promising option for improving results is to avoid the occurrence of pulmonary sepsis by means of timely surgical intervention.

### An analysis of the incidence and subsequent number of surgical interventions of serial rib fractures in Germany for patients below and above the age of 65

ID: 814

**Kategorie: DGT - Thoraxchirurgie bei Alten und Hochbetagten**

Carl Anton Hetzer[1], Jan Beron[1], Klaus-Marius Bastian[1]

[1]*Chirurgische Klinik 4 - Thoraxchirurgie, Rheinland Klinikum Neuss GmbH - Lukaskrankenhaus, Neuss, Deutschland*

**Background:**

Traumatic injuries of the thorax are common and can lead to a significant increase in morbidity and mortality. A substantial amount of traumatic thoracic injuries involve serial rib fractures.

**Materials and methods:**

Using data of ICD-and OPS-codes provided by the German Federal Statistical Office (DESTATIS) for stationary patients, we analysed the incidence of serial rib fractures for patients below and above the age of 65, and the subsequent number of performed surgeries through osteosynthesis of the rib cage. For this analysis, we reviewed data from 2018 to 2020.

**Results:**

The analysis showed an overall decrease of rib fractures from 2018 to 2020, from 31.444 to 29.002. Concerning the two age groups, a decrease of serial rib fractures in patients above the age of 65 became apparent. Conversely, there was an increase for patients below the age of 65. We assume a potential correlation with the Covid-19 pandemic for this development; people above the age of 65 tended so stay indoors more, reducing the risk of thoracic trauma, while those below the age of 65 potentially engaged in more solitary activities with an increased risk of injury, for example through outdoor sports. For the surgical interventions of serial rib fractures, an overall increase from 953 to 1241 between 2018 and 2020 can be seen. However, this only amounts to 5,9% of all OPS-coded osteosyntheses of ribs performed over the same timespan.

**Conclusion:**

We postulate that this percentage is too small, and that a higher number of osteosyntheses for serial rib fractures would lead to an overall better outcome. To validate this assumption, we performed a statistical analysis of the InEK database for serial rib fractures, with a comparison of the actual numbers for osteosyntheses performed in Lukaskrankenhaus Neuss, Rheinland Klinikum Neuss GmbH.

### Looking for an accurate approach for thoracic infection with Actinomyces odontolyticus

ID: 856

**Kategorie: DGT - Thoraxchirurgische Therapie bei seltenen Lungenerkrankungen**

Raffaella Griffo[1], Laura Valentina Klotz[1], Elena Bulut[1], Claus Peter Heußel[2], Michael Allgäuer[3], Martin Eichhorn[1], Cosmas Wimmer[1], Hauke Winter[1]

[1]*Thoraxchirurgie, Thoraxklinik, Heidelberg, Deutschland*, [2]*Radiologie, Thoraxklinik, Heidelberg, Deutschland*, [3]*Pathologie, Thoraxklinik, Heidelberg, Deutschland*

**Background:**

Actinomyces odontolyticus is an anaerobic gram-positive bacterium and is often present in the oral cavity. Actinomycosis is often diagnosed in patients with periodontitis The incidence of actinomycosis is declining.  Pulmonary actinomycosis accounts for less than 15% of all actinomycosis.

Usually, pulmonary actinomycosis gradually grows with air-space consolidation, adjacent pleural thickening, or cavitation in the lung. It is a rare disease with a protracted clinical course. Pulmonary actinomycosis can be misdiagnosed and consequently treated inappropriately. Bronchoscopy or sputum cultures are often insufficient for diagnosis and surgery is in most patients required.

Here we report two clinical cases with progressive pulmonary actinomycosis by A. odontolyticus with their course and resulting complications.

**Materials and methods:**

The first case was a 68-year-old female patient who was admitted to our hospital with a diagnosis of an infracarinal abscess from A. odontolyticus. Long-term antibiotic therapy, repeated interventional bronchoscopies and gastroscopies with endoluminal draining into the oesophagus were carried out for one year. After an antibiotic break of three months, the abscess reoccured with related symptoms of infection and globus sensation. After interdisciplinary discussion we decided to perform a surgical exploration.

The second case involved an 83-year-old female patient who was referred to our hospital with symptoms of dry cough, weight loss, hoarseness, and swallowing problems. Computed tomography (CT) showed a right paratracheal mass with pleural effusion. For an initial suspicion of lung cancer, it was decided to perform a biopsy.

**Results:**

In the first patient, the presumed infra-carinal abscess presented intraoperatively as a bronchogenic cyst. Via right anterolateral thoracotomy, esophageal mobilization with partial extirpation of the cyst and deepithalialisation, resection and reconstruction of the carina and muscle plug insertion using the latissimus dorsi muscle was performed. Postoperative antibiotic therapy and endoscopic monitoring of the anastomosis were carried out. CT-scan after three months showed an uneventful scarred infracarinal healing.

In the second patient, bronchoscopic biopsy of the mediastinal lesion and microbiological analysis of the pleural effusion did not yield any specific findings. Consequently, diagnostic right thoracoscopy was performed. Intraoperatively, a hardening between the superior vena cava and the azygos vein was revealed. The mass was encapsulated with necrotic tissue. Microbiologically, A. odontolyticus was detected from the mediastinal mass. After consultation with the colleagues from the department of infectious diseases, an intravenous antibiotic therapy was initiated according to the antibiogram.
The intravenous antibiotic therapy was complemented by oral antibiotics for several months. The reconvalescence was prolonged due to the intolerance of the patient to the antibiotic therapy which had to be adopted. Furthermore a worsening of a preknown heart failure occurred with bilateral pleural effusions. Fortunately, a dry cough as well as a hoarseness of the voice completely regressed.

**Conclusion:**

Despite antibiotic therapy, patients with pulmonary actinomycosis often experience disease recurrence. Without adequate therapy, the disease progresses chronically with possible invasion of adjacent structures. Long-term antibiotic therapy and follow-up care are therefore essential. Consequently, timely diagnosis and proper management of pulmonary actinomycosis are important because aggressive forms of the disease can develop, especially when these infections are misdiagnosed as a malignant disease or tuberculosis and treatment is delayed. Furthermore a complete restoration of the potential microbiological source (teeth) has to be granted. Physicians should consider these organisms as causative pathogens of lung abscess and acute respiratory failure, especially when the evidence of periodontitis exists.
